# GSA STUDENT CHAPTERS: THE BEGINNING AND HOW TO START A CHAPTER AT YOUR INSTITUTION

**DOI:** 10.1093/geroni/igad104.0603

**Published:** 2023-12-21

**Authors:** Marilyn Gugliucci

**Affiliations:** University of New England College of Osteopathic Medicine, Biddeford, Maine, United States

## Abstract

The Gerontological Society of America (GSA) Student Chapter pilot project was approved by the GSA Board of Directors in December 2020. A GSA representative committee was identified where all GSA member groups were represented, including the selection of three ESPO members who applied to participate. Within a year, a GSA Student Chapter Handbook was written by the committee and reviewed by a GSA Student Advisory Panel (ESPO members who applied and agreed to serve). With the support of the GSA leadership and staff, nine higher education institutions (HEIs) volunteered to pilot a GSA Student Chapter (eight in the U.S. and one in Portugal) during the 2022-23 academic year. The first part of this session will provide information on how to create, implement, and operate a GSA Student Chapter at your HEI. The second part of this session will focus on health professions programs and integrating GSA Student Chapters with other national chapters. With a strong support system and mentoring system within ESPO, health professions programs may benefit from having student chapters integrate from various national organizations. These may provide advancement in learning skills, attitudes and knowledge, along with an extensive network of fellow students and practitioners to support their pre-clinical and clinical health professions education. This enhancement for Student Chapters, GSA with other organization chapters provides national and possibly international organizational connections/networking that will serve students throughout their careers.

